# Knowledge and perception of otosclerosis among women in Saudi Arabia

**DOI:** 10.3389/fpubh.2025.1713127

**Published:** 2025-11-19

**Authors:** Reem Elbeltagy, Shahad Aljwayed, Seham Almutairi, Ghadi Alharbi, Layan Alanzi, Lila Almukhlifi, Rania Alkahtani

**Affiliations:** Department of Health Communication Sciences, College of Health and Rehabilitation Sciences, Princess Nourah bint Abdulrahman University, Riyadh, Saudi Arabia

**Keywords:** otosclerosis, knowledge, perception, Saudi Arabia, women

## Abstract

**Background:**

Otosclerosis is a bone disease of the ear that primarily affects women and can lead to progressive hearing loss. Delays in diagnosis and treatment may occur due to limited awareness of the condition. This study aimed to assess the level of knowledge and perception of otosclerosis among women in Saudi Arabia.

**Methods:**

A cross-sectional study was conducted over 3 months, including 508 Saudi women aged 18 years and above. Data was collected through a structured questionnaire and analyzed using SPSS version 30. The Kruskal–Wallis test was applied to examine differences in knowledge and perception across demographic groups.

**Results:**

Most participants were between 18 and 30 years of age (70.7%), held a bachelor’s degree (63.4%), were students (51.8%), single (66.5%), and residing in the central region (77.0%). The mean knowledge score was 2.44 (SD ± 2.03), indicating low awareness, while the mean perception score was 18.18 (SD ± 3.32), reflecting generally moderate positive attitudes. No statistically significant differences in knowledge or perception were observed across age groups, education levels, or regions (*p* > 0.05).

**Conclusion:**

Public understanding of otosclerosis in Saudi Arabia is inadequate, and reliance on unofficial sources may contribute to misinformation. Efforts to improve health literacy through public campaigns, reliable online resources, and greater involvement of healthcare providers are essential.

## Introduction

Otosclerosis is a pathological bone remodeling disorder that affects the middle and inner ear (oto, “of the ear,” and sclerosis, “abnormal hardening”). It is characterized by abnormal deposition of spongy bone tissue around the stapes, which gradually immobilizes the ossicle by fusing it to the surrounding bony wall, thereby preventing the normal transmission of sound vibrations (1). Clinically, otosclerosis initially manifests as reduced hearing sensitivity to low-frequency sounds. Many patients do not recognize their hearing difficulty in noisy environments, a phenomenon referred to as paracusis Willisii (2). The condition typically causes conductive hearing loss without affecting the tympanic membrane, although cochlear involvement may also occur, resulting in sensorineural hearing loss. Tinnitus is frequently reported, and while its pathophysiology remains unclear, the “exchange hypothesis” suggests that abnormal bone remodeling contributes to its development (3).

Management strategies for otosclerosis primarily aim to halt disease progression rather than cure it. Pharmacological options such as sodium fluoride have been prescribed to slow the remodeling process (4, 5). In selected cases, vitamin D and bioflavonoids have been associated with improved hearing outcomes (6). The most effective and widely adopted intervention is surgical treatment, primarily stapedectomy or stapedotomy, which replaces or bypasses the fixed stapes with a prosthesis to reestablish sound conduction. These procedures have been shown to produce significant and durable improvements in hearing and quality of life (7). Nonetheless, depending on the clinical profile, patient preference, and risk considerations, the use of hearing aids may represent a safer and more suitable alternative for some individuals (8).

From a population perspective, incidence patterns of otosclerosis have shifted significantly over recent decades. A population-based analysis from Olmsted County, Minnesota, revealed an increase in incidence from 8.9 per 100,000 person-years in the 1950s to 18.5 per 100,000 in 1970–1974, followed by a steady decline to 3.2 per 100,000 in 2015–2017. The disease is more prevalent in women than in men, with a ratio of 1.6:1, and it is most commonly diagnosed between the ages of 36 and 55 (9). A British cohort study further confirmed that women have a higher risk of clinical otosclerosis, with a female-to-male ratio of 1.9:1 (10).

From a population perspective, incidence patterns of otosclerosis have shifted significantly, particularly the effects of estrogen on the auditory system. Estrogen has been implicated in promoting pro-inflammatory responses and increased bone turnover, particularly during pregnancy, which may accelerate the onset of otosclerosis (11, 12). Similar inflammatory processes have been observed with hormonal fluctuations during the menstrual cycle and aging. Clinical studies consistently report that a majority of otosclerosis cases occur in women, often manifesting in the fourth decade of life or around pregnancy (13–15).

Beyond its clinical presentation, otosclerosis carries substantial public health implications. If left untreated, it can lead to permanent hearing impairment, tinnitus, and reduced quality of life. Early recognition of symptoms and timely treatment are crucial to preventing long-term disability and promoting better rehabilitation outcomes. However, awareness of the condition among at-risk populations, particularly women, remains largely unexplored. Understanding how women perceive otosclerosis, and the extent of their knowledge about it, is vital for encouraging early consultation, reducing delays in diagnosis, and guiding the design of targeted educational and preventive strategies.

Despite these broader implications, little is known about otosclerosis in Saudi Arabia. While prevalence figures are not well established, hospital-based studies, such as those conducted at King Abdul Aziz University Hospital in Riyadh, have investigated surgical outcomes, indicating the presence of the condition and its clinical significance (16). Moreover, the high rate of consanguinity in the Saudi population is associated with congenital and genetic forms of hearing loss (17–19). Given that otosclerosis has a genetic component, it is plausible that the condition may have a higher incidence in the region (20).

Taken together, these factors underscore the need to investigate awareness of otosclerosis in Saudi Arabia. Considering the limited local research, the established role of hormonal and genetic risk factors, and the disproportionately higher prevalence among women, this study aimed to assess the knowledge and perception of otosclerosis among women in Saudi Arabia. By identifying gaps in awareness, the findings can support healthcare providers and policymakers in developing effective outreach and intervention strategies tailored to women’s health needs.

## Methods

### Study design and setting

A cross-sectional descriptive study was conducted over a three-month period (November 2024 to January 2025) to assess the knowledge and perception of otosclerosis among women in Saudi Arabia. The study targeted adult Saudi females aged 18 years and above residing in various regions of the country. Male participants and non-Saudi nationals were excluded.

### Sample size

The required sample size was calculated using the EPI Info online calculator. Assuming a prevalence of 50% (to maximize sample representativeness) and an infinite population, with a 95% confidence level and 5% margin of error, the minimum sample required was 384 participants.

### Instrument

Data were collected using a self-administered electronic questionnaire, developed in Arabic, consisting of 24 items. The questionnaire was structured into three sections:

1. **Demographics**: Age, education level, occupation, marital status, and region of residence.
2. **Knowledge**: Multiple-choice questions assessing participants’ knowledge of otosclerosis.
3. **Perception**: Statements related to participants’ perception of otosclerosis, rated on a 5-point Likert scale (1 = strongly disagree, 5 = strongly agree).

Content validity of the instrument was evaluated by three experts in audiology, otology and public health to ensure clarity, relevance, and comprehensiveness of the items. Their feedback was incorporated to refine the content and structure of the questionnaire. Although content validation is often conducted with 5–10 experts, evidence suggests that three or more domain experts can provide sufficient evaluation when they have relevant expertise and familiarity with the construct being assessed (21, 22). The scale validity index (S-CVI/Ave) was 1.0, indicating excellent agreement among experts. Reliability was assessed using Cronbach’s *α* test. Cronbach’s α value was 0.88 which indicates very good reliability.

### Data collection and analysis

Responses were collected electronically through Google Forms and subsequently exported into IBM SPSS Statistics software, version 30 (IBM Corp. 2023 Armonk, NY) for analysis. Descriptive statistics, including frequencies and percentages, were used to summarize demographic characteristics. Means and standard deviations were calculated for knowledge and perception scores.

### Methodology for analyzing knowledge and perception

To analyze the survey data, two distinct scoring methods were used for the knowledge and perception sections.

### Knowledge score

The knowledge section consisted of nine items, was graded by assigning 1 point for each correct answer. The total knowledge score was calculated by summing the points from all nine items, resulting in a possible score range of 0 to 9. A higher score indicated a greater level of knowledge.

- **Excellent Knowledge**: Scores above 75% (greater than 6.75 points)
- **Good Knowledge**: Scores between 50 and 75% (4.5 to 6.75 points)
- **Poor Knowledge**: Scores below 50% (less than 4.5 points)

### Perception score

The perception section used a 5-point Likert scale (1 = Strongly Disagree to 5 = Strongly Agree) for five items. The total perception score was calculated by summing the scores of all five items, with a possible range from 5 to 25. A higher score indicated a more positive perception of otosclerosis.

- **Positive Perception**: Scores above 75% (greater than 18.75 points)
- **Moderate Positive Perception**: Scores between 50 and 75% (12.5 to 18.75 points)
- **Negative Perception**: Scores below 50% (less than 12.5 points)

The Kruskal–Wallis test, a non-parametric alternative to one-way ANOVA, was employed to examine differences in mean responses across variables with three or more categories. A *p*-value < 0.05 was considered statistically significant.

### Ethical considerations

This study was approved by the Institutional Review Board of Princess Nourah bint Abdulrahman University (Approval No. 24–0868). Electronic informed consent was obtained from all participants prior to survey completion. Participants were informed about the study objectives and assured that their responses would remain anonymous and confidential. No identifying information were collected.

## Results

A total of 508 women participated in the survey. Among them, 38.0% were aged 21–30 years, while 32.7% were aged 18–20 years. More than half of the participants (51.8%) were students, followed by 22.6% who were employees. The majority were single, held a bachelor’s degree, and resided in the central region of Saudi Arabia (66.5, 63.4, and 77.0%, respectively). The detailed demographic characteristics of the participants are presented in Table 1.

**Table 1:** Demographic characteristics of participants.

Variable	Category	N	%
Age in year	18–20	166	32.7%
	21–30	193	38.0%
	31–40	66	13.0%
	41–50	59	11.6%
	51–60	24	4.7%
Education level	Elementary school certificate	2	0.4%
	Middle School certificate	10	2.0%
	High school diploma or equivalent	149	29.3%
	Bachelor’s degree	322	63.4%
	Postgraduate degree	25	4.9%
Occupation	Housewife	56	11.0%
	Student	263	51.8%
	Unemployed	62	12.2%
	Retired	12	2.4%
	Employee	115	22.6%
Marital status	Single	338	66.5%
	Married	155	30.5%
	Have been married before	15	3.0%
Region	Central region	391	77.0%
	Eastern region	47	9.3%
	Western region	18	3.5%
	Northern region	20	3.9%
	Southern region	32	6.3%

With regard to knowledge, the mean score was 2.44 (SD ± 2.03). The item with the highest correct response was “Who has a higher risk of getting otosclerosis?” which 48.4% of participants answered correctly, whereas the lowest was “What are the factors that may contribute to the development of otosclerosis?” with only 10.8% correct responses. A detailed distribution of knowledge responses is presented in Table 2.

**Table 2:** Participants’ knowledge about otosclerosis.

Statement	Correct	Incorrect
Have you ever heard of the term “otosclerosis.”?	178 (35.0%)	330 (65.0%)
Who has a higher risk of getting otosclerosis?	246 (48.4%)	262 (51.6%)
Otosclerosis affects the bones of?	193 (38.0%)	315 (62.0%)
What are the symptoms you know about otosclerosis?	60 (11.8%)	448 (88.2%)
What are the factors that may contribute to the development of otosclerosis?	55 (10.8%)	453 (89.2%)
Otosclerosis is diagnosed through:	104 (20.5%)	404 (79.5%)
Can otosclerosis be treated?	198 (39.0%)	310 (61.0%)
If your answer is yes, what treatment options do you know?	118 (23.2%)	390 (76.8%)
What is the importance of early diagnosis of otosclerosis?	90 (17.7%)	418 (82.3%)
Community Knowledge About Otosclerosis (Mean ±STD)	2.44 ± 2.03	

In terms of perception, the mean score was 18.18 (out of 25) with a relatively low standard deviation of ±3.32 suggests a moderately positive perception of otosclerosis among respondents. The strongest agreement was observed for the statement on the need to raise awareness about otosclerosis, with 61.6% of participants strongly agreeing. In contrast, the highest neutrality was reported for the statement concerning whether individuals with otosclerosis receive adequate support, with 52.2% selecting “neutral.” The detailed perception responses are presented in Table 3.

**Table 3:** Participants’ perception about otosclerosis.

Statement	Strongly disagree	Disagree	Neutral	Agree	Strongly agree
I believe that otosclerosis is a common condition.	32 (6.3%)	136 (26.8%)	233 (45.9%)	69 (13.6%)	38 (7.5%)
I think otosclerosis may affect a person’s daily life.	12 (2.4%)	18 (3.5%)	126 (24.8%)	181 (35.6%)	171 (33.7%)
I believe caregivers play a crucial role in raising patients’ awareness about otosclerosis.	23 (4.5%)	32 (6.3%)	111 (21.9%)	139 (27.4%)	203 (40.0%)
I believe that people with otosclerosis receive adequate support.	28 (5.5%)	78 (15.4%)	265 (52.2%)	91 (17.9%)	46 (9.1%)
I believe there is a need to raise awareness about otosclerosis.	17 (3.3%)	15 (3.0%)	65 (12.8%)	98 (19.3%)	313 (61.6%)
perception about otosclerosis (Mean ±STD)	18.18 ± 3.32				

When asked about their sources of information on otosclerosis, friends and family were cited most frequently (12.4%), whereas awareness campaigns were the least reported source (2.2%). Regarding preferred methods for receiving further information, social media was identified as the most favored option (76.2%), while brochures were the least preferred, chosen by only 15.4% of respondents. These findings are summarized in Table 4.

**Table 4:** Participants source of information about otosclerosis.

Statement	Source	N	%
What was your source of information? (178 participants who have heard about otosclerosis)	Internet	59	11.6%
	Friends and family	63	12.4%
	In educational institutes (school or university)	53	10.4%
	Doctor	22	4.3%
	Media	52	10.2%
	Awareness campaign	11	2.2%
What is your preferred way to receive more information about otosclerosis?	Social relationships	105	20.7%
	Social media	387	76.2%
	Text messages	125	24.6%
	Workshops	85	16.7%
	Public events	145	28.5%
	Brochures	78	15.4%

Participants were also asked about the perceived impact of otosclerosis on daily life. Nearly half (47.4%) indicated that work was the most affected domain. Because these questions allowed multiple responses, the findings reflect a range of opinions rather than exclusive categories. The detailed distribution of perceived impacts is presented in Table 5.

**Table 5:** Aspects of life affected by otosclerosis.

If your answer shows agreement to the statement “I think otosclerosis may affect a person’s daily life,” which aspects do you think might be affected?”	N	%
Social relationships	186	36.6%
Difficulty communicating	197	38.8%
Social isolation	136	26.8%
Work	241	47.4%
Education	185	36.4%
Entertainment activities	166	32.7%
Mental health	202	39.8%
I do not know	106	20.9%

Finally, the Kruskal–Wallis test revealed no statistically significant differences in knowledge or perception scores across education levels (knowledge: *p* = 0.190; perception: *p* = 0.058), age groups (knowledge: *p* = 0.184; perception: *p* = 0.169), or regions (knowledge: *p* = 0.793; perception: *p* = 0.174). The detailed results of these comparisons are shown in Table 6.

**Table 6:** Association between knowledge, perception and socio-demographic characteristics.

Variable			N	Mean rank	Kruskal-Wallis H	df	*p*-value
Age in year	Knowledge of Otosclerosis	18–20	166	232.2	6.214	4	0.184
		21–30	193	261.38			
		31–40	66	271.16			
		41–50	59	268.17			
		50–60	24	273.96			
	Perception of otosclerosis	18–20	166	267.86	6.441	4	0.169
		21–30	193	262.44			
		31–40	66	230.98			
		41–50	59	226.53			
		50–60	24	231.73			
Level of education	Knowledge of Otosclerosis	Elementary school certificate	2	421.25	6.132	4	0.190
		Middle School certificate	10	272.60			
		High school diploma or equivalent	149	235.56			
		Bachelor’s degree	322	261.55			
		Postgraduate degree	25	256.02			
	Perception of otosclerosis	Elementary school certificate	2	504.00	9.143	4	0.058
		Middle School certificate	10	335.60			
		High school diploma or equivalent	149	252.75			
		Bachelor’s degree	322	251.78			
		Postgraduate degree	25	247.56			
Region	Knowledge of Otosclerosis	central region	391	250.95	1.689	4	0.793
		eastern region	47	258.22			
		western region	18	269.36			
		north region	20	256.45			
		south region	32	282.86			
	Perception of otosclerosis	central region	391	253.87	6.360	4	0.147
		eastern region	47	217.33			
		western region	18	259.83			
		north region	20	294.70			
		south region	32	288.61			

## Discussion

Since otosclerosis has a higher prevalence among women (23), this study assessed knowledge and perception of otosclerosis among women in Saudi Arabia. To achieve this, a questionnaire was distributed across different age groups, educational backgrounds, and regions.

With regard to demographics, most participants were young, with the largest group (38.0%) aged 21–30 years and 32.7% aged 18–20 years. This finding is consistent with Almalki et al. who reported increasing engagement of younger and female populations in Saudi healthcare utilization. More than half of the participants were students (51.8%), which reflects the young sample, and the majority (63.4%) held a bachelor’s degree. Participants were mainly from the central region, which may be explained by the concentration of the population in Riyadh, the capital and largest city in Saudi Arabia. However, this overrepresentation of the central region, together with the predominance of younger participants, may limit the generalizability of the findings to the wider Saudi population (24).

The study revealed a low level of knowledge about otosclerosis. The mean knowledge score was only 2.44 (SD = 2.03), and just 35% of participants had ever heard of the condition. Despite the relatively young and well-educated sample, most participants were unfamiliar with the disease. Many participants also held misconceptions. For example, more than half (51.6%) were incorrect about who is at higher risk of otosclerosis, 62% did not know which bones are affected, and over 80% were unaware of the symptoms or contributing factors. Knowledge about diagnosis and treatment was similarly poor. These findings highlight a major public health gap. They are in line with previous studies in Saudi Arabia reporting poor or fair awareness of ear health. For example, Alshehri et al. (25) found that individuals with a family history of hearing loss had better knowledge, and Alsaawi et al. (26) reported low awareness of hearing loss management in Buraidah. Together, these studies suggest that while the public may be somewhat aware of general hearing problems, knowledge of specific conditions such as otosclerosis is lacking.

In contrast to the low knowledge levels, perception toward otosclerosis was generally positive. The mean perception score was 18.18 (SD = 3.32). A large majority of participants agreed that otosclerosis has a significant impact on daily life, and 67.4% emphasized the role of caregivers in raising awareness, reflecting support for a community-based health education approach. Moreover, 80.9% agreed that more awareness is needed, indicating a demand for better information. However, when asked whether patients receive adequate support, over half of the participants (52.2%) were neutral, reflecting either limited knowledge of available resources or uncertainty about patient care. Similar discrepancies between knowledge and perception have been reported in other studies. For instance, Alshehri et al. (25) noted that despite fair knowledge of ear health, participants strongly supported early intervention, while Albulayhid et al. (27) found poor awareness of otitis externa but positive attitudes toward seeking professional care.

Sources of information also revealed important insights. Most participants relied on informal sources, such as friends and family (12.4%), the internet (11.6%), and media (10.2%), while only 4.3% reported receiving information from doctors and just 2.2% from awareness campaigns. This aligns with Al-Muammar et al. (28), who found that informal sources dominate health information in Saudi Arabia, as well as global trends. The reliance on non-professional sources may explain the low knowledge levels observed. Supporting this, La Rosa et al. (29) showed that online resources about otosclerosis often have low readability and variable quality, making them unreliable for patient education. In terms of preferred methods for future information, the majority (76.2%) favored social media, followed by public events (28.5%) and text messages (24.6%). Traditional channels such as brochures were less preferred. These findings suggest that public health campaigns should prioritize digital platforms to effectively reach younger populations, while also strengthening communication between doctors and patients.

Regarding the perceived impact of otosclerosis, nearly half of the participants (47.4%) believed that work would be the most affected domain. This aligns with Emmett and Francis (30), who found that individuals with hearing loss were almost twice as likely to be unemployed as those with normal hearing. Participants also reported potential effects on mental health (39.8%) and social relationships (36.6%). West (31) highlighted the impact of hearing impairment on marital relationships, particularly increased depressive symptoms in husbands of women with hearing loss, although other studies such as Ask et al. (32) found weaker associations. Lima et al. (33) further showed that tinnitus, common in otosclerosis, can significantly impair daily functioning, especially among women and older adults. These findings reinforce the wide-ranging impact of otosclerosis on quality of life.

Finally, no statistically significant differences were observed in knowledge or perception across age, education level, or region. Knowledge did not vary significantly with age (*p* = 0.184) or perception (*p* = 0.169). This contrasts with Alshehri et al. (25) and Bedaiwi et al. (34), who reported higher awareness among older adults, suggesting that the effect of age may differ depending on the condition or context. Similarly, education level was not associated with differences in knowledge (*p* = 0.190) or perception (*p* = 0.058). Previous studies have shown that higher education is often linked to better health knowledge (35), but this was not evident here, indicating that awareness of otosclerosis may not be influenced by formal education. Regional comparisons also showed no significant differences (knowledge: *p* = 0.793; perception: *p* = 0.174). This finding is consistent with Alsudays et al. (36), who noted that while parents in Qassim held positive attitudes toward audiology services, their understanding of hearing impairment remained limited.

In summary, this study highlights a substantial gap in knowledge about otosclerosis among women in Saudi Arabia, despite generally moderate positive perceptions and recognition of its impact. While the findings provide useful insights, they should be interpreted with caution given the predominance of young participants and the overrepresentation of the central region. Future studies should examine the relationship between awareness levels and clinical outcomes, including early diagnosis and treatment adherence, assess the effectiveness of targeted educational interventions, and include more diverse demographic groups to strengthen generalizability.

## Limitations

This study has some limitations that should be considered when interpreting the findings. First, the sample was predominantly young women and largely drawn from the central region, which may limit the generalizability of the results to the wider Saudi population. Second, the use of a self-administered electronic survey may have introduced selection bias, as individuals with internet access and higher education were more likely to participate. Third, cross-sectional design captures knowledge and perception at a single point in time, and therefore does not allow for assessment of changes over time or causal relationships. Despite these limitations, the study provides valuable baseline data on awareness of otosclerosis among women in Saudi Arabia and highlights areas for future research.

## Conclusion

In Saudi Arabia, the public’s understanding of otosclerosis remains insufficient, with much of the available information obtained from unofficial sources that may spread misinformation. Efforts should therefore be directed toward improving health literacy through well-designed public campaigns, the development of reliable online resources, and greater involvement of healthcare providers in patient education. Leveraging social media platforms responsibly and presenting information in clear, accessible formats can help ensure that accurate knowledge reaches the public. By strengthening awareness and facilitating timely consultation, individuals with otosclerosis may benefit from earlier diagnosis, more effective treatment, and ultimately an improved quality of life.

## Data Availability

The raw data supporting the conclusions of this article will be made available by the authors, without undue reservation.
