# Supplementary material for: Knowledge and perception of otosclerosis among women in Saudi Arabia
Source: Front Public Health. 2025 Nov 19;13:1713127. doi: 10.3389/fpubh.2025.1713127 (PMC12672442; doi:10.3389/fpubh.2025.1713127)
Supplement: Supplementary file 1 [file Data_Sheet_1.pdf]

Appendix A

Questionnaire

knowledge and Perception of Otosclerosis Among Women In Saudi Arabia

Demographic Information:

1-Age:
- 18 -20
- 21-30
- 31-40
- 41-50
- 51-60

2-Education level:
- Elementary school
- Middle School
- Some high school
- High school diploma or equivalent
- Some college
- Bachelor's degree
- Graduate degree

3- occupation
- Employee
- Unemployed
- Retired
- Student

4- What is your marital status:
- married
- single
- widowed

5- In which region of Saudi Arabia do you live?
- central
- eastern
- western
- north
- south

Community Knowledge About Otosclerosis:

1- Have you ever heard of the term "otosclerosis."?
- Yes
- No

2- If yes, what was your source of information?
- Doctor
- Media
- internet
- Friends and family
- Awareness campaign
- in educational institutes (school or university)

3- Who has a higher risk of getting otosclerosis?
- Females
- Males
- Females and Males equally

4- Otosclerosis affects the bones of?
- Hand
- Foot
- Knee
- Ear
- Head
- I don't know

5- What are the symptoms you know about otosclerosis?
- Hearing loss
- Ringing in the ear
- Dizziness
- Difficulty understanding speech
- Ear pain
- Increasing ear wax
- Pain in the hand
- Pain in the foot
- Headache
- Pain in the head
- I don't know

6- What are the factors that may contribute to the development of otosclerosis?
- Hereditary
- Aging
- Infection
- Exposure to noise
- Pregnancy
- Breastfeeding
- Contraceptive drugs
- I don't know

7- Otosclerosis is diagnosed through:
- Laboratory tests
- Clinical examination
- Imaging (x rat, CT scans)
- Hearing assessments
- Vestibular Assessments

- I don’t know

8- Can otosclerosis be treated?

- Yes
- No
- I don’t know

9- If your answer is yes, what treatment options do you know?

- Drugs
- Surgery
- Hearing Aids
- I don’t know

10- What is the importance of early diagnosis of otosclerosis?

- It prevents the disease from worsening
- Providing better treatment options
- Improving quality of life
- I don’t know

Measuring the level of perception about otosclerosis:

1- I believe that otosclerosis is a common condition.

- Strongly Agree
- Agree
- Neutral
- Disagree
- Strongly Disagree

2- I think otosclerosis may affect a person's daily life.

- Strongly Agree
- Agree
- Neutral
- Disagree
- Strongly Disagree

3- If you answer with 'Agree' or 'Strongly agree,' which aspects do you think might be affected?"

- Social relationships
- Work
- Education
- Entertainment
- Difficult communication
- Social isolation
- Mental health
- I don’t know

4- I believe caregivers play a crucial role in raising patients' awareness about otosclerosis.

- Strongly Agree
- Agree
- Neutral
- Disagree

5- I believe that people with otosclerosis receive adequate support.

- Strongly Agree
- Agree
- Neutral
- Disagree
- Strongly Disagree

6- I believe there is a need to raise awareness about otosclerosis.

- Strongly Agree
- Agree
- Neutral
- Disagree
- Strongly Disagree

7- What is your preferred way to receive more information about otosclerosis?

- Social media
- SMS messages
- Brochures
- Workshops
- Community meetings
- public events
